# Emerging Technologies for the Management of Type 1 Diabetes in Pregnancy

**DOI:** 10.1007/s11892-018-0973-9

**Published:** 2018-01-30

**Authors:** Jennifer M. Yamamoto, Helen R. Murphy

**Affiliations:** 1 Department of Medicine, Division of Endocrinology and Metabolism, University of Calgary, Calgary, AB Canada; 2 Cambridge University Hospitals NHS Foundation Trust, Cambridge, UK; 3 Women’s Health Academic Centre, Division of Women’s and Children’s Health, King’s College London, London, UK; 4 Norwich Medical School, University of East Anglia, Floor 2, Bob Champion Research and Education Building, James Watson Road, Norwich Research Park, Norwich, NR4 7UQ UK

**Keywords:** Diabetes in pregnancy, Technology, Continuous glucose monitoring, Insulin pump, Closed loop, Artificial pancreas

## Abstract

**Purpose of Review:**

The purpose of the study is to discuss emerging technologies available in the management of type 1 diabetes in pregnancy.

**Recent Findings:**

The latest evidence suggests that continuous glucose monitoring (CGM) should be offered to all women on intensive insulin therapy in early pregnancy. Studies have additionally demonstrated the ability of CGM to help gain insight into specific glucose profiles as they relate to glycaemic targets and pregnancy outcomes. Despite new studies comparing insulin pump therapy to multiple daily injections, its effectiveness in improving glucose and pregnancy outcomes remains unclear. Sensor-integrated insulin delivery (also called artificial pancreas or closed-loop insulin delivery) in pregnancy has been demonstrated to improve time in target and performs well despite the changing insulin demands of pregnancy.

**Summary:**

Emerging technologies show promise in the management of type 1 diabetes in pregnancy; however, research must continue to keep up as technology advances. Further research is needed to clarify the role technology can play in optimising glucose control before and during pregnancy as well as to understand which women are candidates for sensor-integrated insulin delivery.

## Introduction

Pregnancies in women with type 1 diabetes continue to be at increased risk of potentially serious complications [1]. The risk of these complications can be attenuated by tight glycaemic control preconception and throughout pregnancy [2, 3]. While tight glycaemic control is central to the management of type 1 diabetes in pregnancy, achieving the targets necessary to reduce pregnancy-related complications remains challenging. Many physiologic changes throughout pregnancy as well as other unique circumstances such as labour and delivery and the postpartum period make frequent insulin adjustments and close follow-up necessary [4, 5].

Despite specialised interdisciplinary clinics, and advances in the treatment of women with type 1 diabetes, a large contemporary cohort study in the UK found that most women did not achieve optimal glycaemic control [6••]. More specifically, only 16 and 40% of women with type 1 diabetes in early and late pregnancy respectively met guideline-suggested haemoglobin A1c (HbA1c) targets of less than 48 mmol/mol (6.5%) [6••]. This nationwide study did note improvement in some pregnancy outcomes, namely a 2.5-fold reduction in stillbirths; however, one in two babies still experienced complications related to maternal hyperglycaemia, namely large for gestational age, preterm delivery and/or admission to neonatal intensive care units. It also highlights the substantial contribution of between clinic variation suggesting much room for improvement in the way we care for women with type 1 diabetes before and during pregnancy. The use of technology in the treatment of women with diabetes may help bridge the gap between current and optimal glycaemic control in pregnancy with the goal of improving outcomes for mother and infant.

This review will discuss emerging technologies available in the management of type 1 diabetes in pregnancy and their effect on glycaemic control and pregnancy outcomes. More specifically, we will review the use of continuous glucose monitoring (CGM) technologies, the insulin pump and sensor-integrated insulin delivery in care of women with type 1 diabetes in pregnancy.

## Continuous Glucose Monitoring

CGM uses a sensor inserted subcutaneously to record glucose concentrations in the interstitial fluid generating almost 300 glucose measurements per day. It communicates the measured glucoses to a mobile phone, smartwatch or stand-alone receiver device via Bluetooth. The CGM sensor, which is disposable, is typically changed weekly, and the transmitter duration is approximately 12 months depending on the system. The accuracy of CGM has improved substantially over the past 5–10 years with some systems now considered accurate enough to enable pre-meal bolusing without the need for confirmation with capillary glucose testing. However, most devices, other than the Flash glucose monitor, still require calibration with capillary glucose testing two to four times a day [7]. The Flash glucose monitoring system is factory calibrated and is considered a replacement for capillary glucose testing rather than a continuous glucose monitoring system as it lacks hypoglycaemia and hyperglycaemia alerts and alarms and is not integrated with insulin delivery.

CGM yields rich glucose data that can be used to manage insulin in real time, examine glycaemic trends, and study glucose metabolism [8]. Outside of pregnancy, CGM has been shown to improve glucose control in selected populations who are motivated to wear the device regularly (at least 6 days per week) [9]. Further evidence suggests that the use of CGM can also reduce hypoglycaemia [10]. Various studies have highlighted both benefits and drawbacks of the use of this tool in women with type 1 diabetes in pregnancy.

### CGM and Pregnancy Outcomes

HbA1c is an important tool used to monitor glycaemic control and assess the maternal-infant level of risk; however, it has established limitations in pregnancy. Firstly, HbA1c is influenced by physiologic changes in pregnancy such as increased red cell turnover in early gestation, which is known to change as pregnancy progresses [11, 12]. Secondly, because HbA1c levels are physiologically lower in early to mid-pregnancy (16–20-week gestation), this can give false reassurance to patients and clinicians. Thirdly, HbA1c represents an average measure of glycaemic control but does not yield detailed information on the pattern of daily glycaemic excursions which is needed to guide therapy adjustments. In contrast, CGM gives clinicians and researchers detailed information regarding time spent in the recommended target range as well as patterns of glucose fluctuations that may be used to optimise diet, lifestyle and insulin adjustments. It also can alert users to impending hypoglycaemia or hyperglycaemia allowing them to take earlier corrective action and therefore minimise out-of-range excursions. The immediate feedback from CGM may deepen the user’s understanding of what influences their glucose profile and may encourage them to alter diet, activity and insulin adjustment behaviours.

Four studies, including two recent studies, have examined the use of CGM in the management of diabetes in pregnancy (Table 1). Murphy et al. studied 71 women, including 46 with type 1 diabetes, in an open-label, randomised controlled trial of patients in the UK [13]. Women randomised to receive CGM wore it between 8 and 32-week gestation for up to 7 days every 4–6 weeks. Both the women and their healthcare providers were masked to the CGM information until it was reviewed at their clinic visit. Authors found that the use of CGM was associated with a lower mean HbA1c level between weeks 32 and 36 (5.8 vs. 6.4%; *p* = 0.007) and reduced risk of macrosomia (odds ratio [OR] 0.36 [95% confidence interval [CI] 0.13 to 0.98]; *p* = 0.05).

**Table 1:** Studies examining the use of continuous glucose monitoring in pregnancy

First author, year	Country	Number	Gestational age at randomisation	Intervention	Main results of CGM vs. control	
					Maternal outcomes	Neonatal outcomes
Feig et al. 2017 [20••]	Canada, UK, Spain, Italy, Ireland and USA	325 (all with type 1 diabetes; 110 pre-pregnancy, 215 pregnant)	< 14 weeks or planning pregnancy	Continuous real-time CGM	Lower HbA1c (mean difference − 0.2% [95% CI 0.34, − 0.03%]; *p* = 0.02) More time in target (68 vs. 61%; *p* = 0.003) Less time hyperglycemic (27 vs. 32%; *p* = 0.03)	Less large for gestational age (OR 0.51 [95% CI 0.28, 0.90]; *p* = 0.02) Less admissions to neonatal intensive care units (OR 0.48 [95% CI 0.26, 0.86]; *p* = 0.02) Fewer episodes of neonatal hypoglycaemia (OR 0.45 [95% CI 0.22, 0.89]; *p* = 0.02)
Voormolen et al. 2017^a^ [18]	Netherlands	304 (type 1 diabetes = 109, type 2 diabetes = 83, gestational diabetes = 112)	Type 1 and type 2 diabetes: < 16 weeks Gestational diabetes: < 30 weeks	Intermittent masked CGM for 5–7 days every 6 weeks	Less preeclampsia in the CGM group (percent and *p* value not reported)	No difference in macrosomia between the two groups (relative risk 0.99 [95% CI 0.76, 1.28])
Secher et al. 2013 [14]	Denmark	154 (type 1 diabetes = 123, type 2 diabetes = 31)	< 14 weeks	Intermittent real-time CGM for 6 days at 8, 12, 21, 27 and 33 weeks	At 33 weeks, HbA1c in each group was similar (6.1 vs. 6.1%, *p* = 0.39)	No difference in large for gestational age (45 vs. 34%, *p* = 0.19)
Murphy et al. 2008 [13]	UK	71 (type 1 diabetes = 46, type 2 diabetes = 25)	< 8 weeks	Masked CGM for 7 days every 4–6 weeks	Lower mean HbA1c between weeks 32 and 36 (5.8 vs. 6.4%; *p* = 0.007)	Reduced risk of macrosomia (OR 0.36 [95% CI 0.13 to 0.98]; *p* = 0.05)

^a^Protocol and abstract only

In contrast, Secher et al. did not find evidence of an improvement in glycaemic control or pregnancy outcomes in their larger study of 154 women (123 women with type 1 diabetes and 31 women with type 2 diabetes) [14]. In this open-label study, women were randomised to receive standard care with or without supplementary real-time CGM. If randomised to CGM, women were asked to wear the device for 6 days on five occasions between 8 and 33-week gestation. The women randomised to CGM reported substantial burdens such as discomfort with the device, disturbed sleep and technical challenges, with only 49 women (64%) using it as per study protocol and only 5 women (7%) wearing CGM for at least 60% of the time. There was no significant difference in their primary outcome large for gestational age (45 vs. 34%, *p* = 0.19), and at 33 weeks, the HbA1c in each group was similar (6.1 vs. 6.1%, *p* = 0.39) [14, 15]. Unfortunately, the directly observed CGM data was not reported for the control group in either of these studies. A subsequent Cochrane review of glucose monitoring in diabetes pregnancy concluded that further high-quality randomised trials evaluating the maternal, neonatal and psychosocial outcomes were needed [16].

Two contemporary studies “Effectiveness of Continuous Glucose Monitoring During Diabetic Pregnancy” (GlucoMOMS trial) and “Continuous Glucose Monitoring in Women with Type 1 Diabetes in Pregnancy Trial” (CONCEPTT) examined the use of CGM in pregnancies complicated by diabetes. GlucoMOMS trial was a multicentre open-label randomised controlled trial with a concurrent cost-effectiveness study [17]. Women with type 1 diabetes, type 2 diabetes or gestational diabetes requiring insulin were randomised to intermittent masked CGM for 5–7 days every 6 weeks vs. standard of care. It included 304 women with approximately equal numbers with type 1, type 2 and gestational diabetes, from over 20 sites in the Netherlands. They found no significant difference in macrosomia between the two groups (relative risk 0.99 [95% CI 0.76, 1.28]) but did find less preeclampsia in the CGM group (abstract only) [18]. The inclusion of women with different degrees of glycaemic disturbance and very different rates of macrosomia, ranging from just above normal in gestational diabetes to one in two in type 1 diabetes offspring, makes it difficult to comment definitively on whether there is a role for CGM in any specific type of diabetes.

**Fig. 1:**
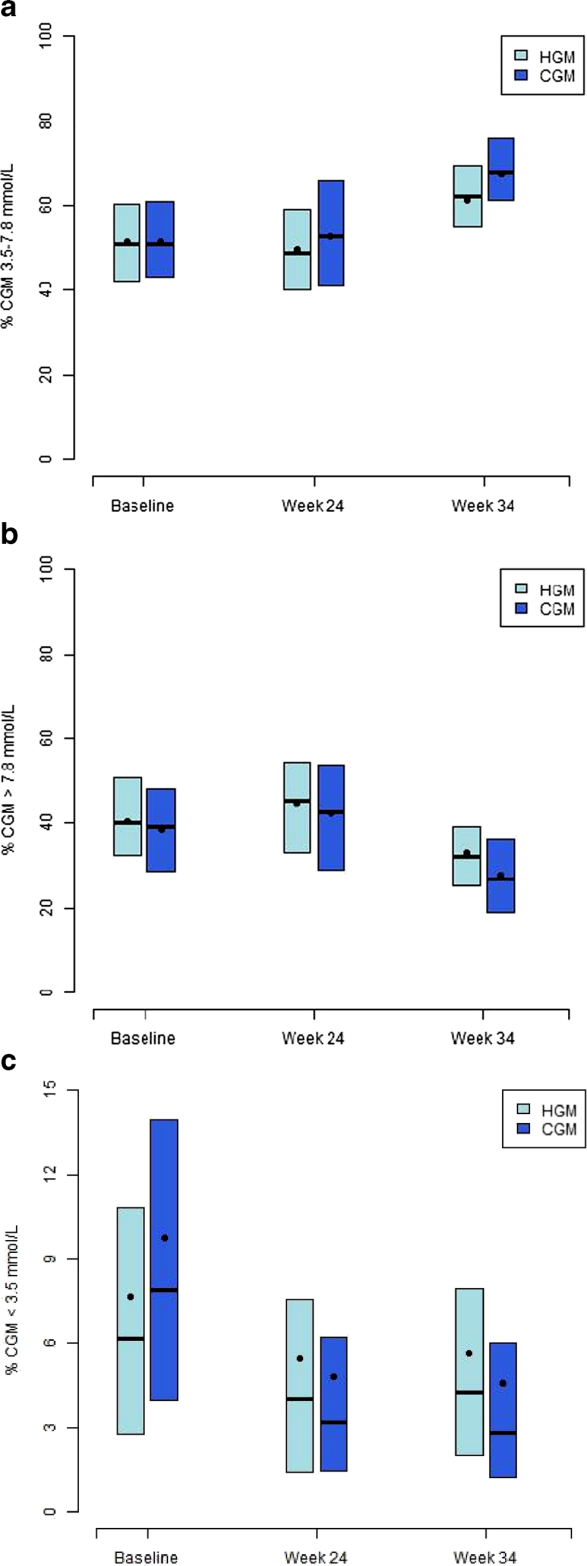
Continuous glucose monitoring (CGM) measures from CONCEPTT. **a** Time in target range 3.5–7.8 mmol/L (70–140 mg/dL). The home glucose monitoring (HGM) group spent 52% time in target at baseline (12.5 h/day) rising to 61% (14.6 h/day) at 34 weeks. The CGM group spent 52% time in target at baseline (12.5 h/day) rising to 68% (16.3 h/day) at 34-week gestation; *p* = 0.003 for between group difference. **b** Time spent hyperglycaemic > 7.8 mmol/L (140 mg/dL). The HGM group spent 40% time hyperglycaemic at baseline (9.6 h/day) reducing to 32% (7.7 h/day) at 34 weeks. The CGM group spent 39% time in target at baseline (9.4 h/day) reducing to 27% (6.5 h/day) at 34-week gestation; *p* = 0.03 for between group difference. **c** Time spent hypoglycaemic < 3.5 mmol/L (70 mg/dL). The HGM group spent 8% time hyperglycaemic at baseline (1.9 h/day) reducing to 4% (1.0 h/day) at 34 weeks. The CGM group spent 6% time in target at baseline (1.4 h/day) reducing to 3% (0.7 h/day) at 34-week gestation; *p* = 0.10 for between group difference

CONCEPTT was a multicentre, open-label trial where women type 1 diabetes preconception or < 14-week gestation was randomised to capillary glucose monitoring with and without real-time CGM [19]. In contrast to previous trials of intermittent retrospective or real-time CGM, in CONCEPTT, women were recommended to use real-time CGM continuously from randomisation until delivery or 24 weeks after randomisation in the pre-pregnancy group who did not conceive. It recruited 325 women (110 pre-pregnancy, 215 pregnant) from 31 sites in Canada, the UK, Spain, Italy, Ireland and the USA making it the largest randomised trial in type 1 diabetes pregnancy [20••]. The recently reported results from CONCEPTT found that while pregnant women randomised to CGM had only slightly lower HbA1c levels (mean difference − 0.2% [95% CI 0.34, − 0.03%]; *p* = 0.02), they spent an additional 100 min per day in the recommended glucose control range (CGM time in target 70–140 mg/dL range 68 vs. 61%; *p* = 0.003) with 72 min less hyperglycaemia (27 vs. 32%; *p* = 0.03) at 34-week gestation (Fig. 1). Importantly, the improvements in glucose control were equal for women using multiple daily injections (MDI) and women using insulin pumps. There were no substantial between group differences in patient-reported psychosocial outcomes. Questionnaires assessing maternal glucose monitoring and CGM satisfaction indicated overall favourable ratings. Hypoglycaemia avoidance behaviours decreased over time in women using CGM, but stayed constant over time in the control group.

However, the most striking finding was not on maternal glycaemia but rather on neonatal outcomes, with significant reductions in the rate of large for gestational age birthweight (OR 0.51 [95% CI 0.28, 0.90]; *p* = 0.02), admission to neonatal intensive care units (OR 0.48 [95% CI 0.26, 0.86]; *p* = 0.02) and episodes of neonatal hypoglycaemia requiring intravenous dextrose infusion (OR 0.45 [95% CI 0.22, 0.89]; *p* = 0.02). Furthermore, the infants of mothers randomised to CGM had a 1-day shorter total length of hospital stay (3.1 vs. 4.0 days; *p* = 0.009). The numbers needed to treat with CGM to prevent one complication are six for both neonatal intensive care admission and large for gestational age and eight for neonatal hypoglycaemia. The authors therefore conclude that CGM should now be offered to all women with type 1 diabetes during the first trimester. Further health economic evaluations are planned to determine whether the additional costs of CGM are offset by the reductions in neonatal care and shorter length of hospital stay. The authors did not find convincing evidence to decide whether CGM should be offered to women before pregnancy. As only a minority of women (15% in UK nationwide study) achieve target glucose control in early pregnancy, further research is needed in a larger sample of pre-pregnant women, preferably over a longer duration (52 weeks) to determine whether or not there is a role for CGM in women planning pregnancy.

### Using CGM to Gain Insight into Glucose Pathophysiology

The detailed information provided by CGM is improving our understanding of various aspects of glucose control and in some cases deepening our understanding of the complex relationship between glucose and pregnancy outcomes. Law et al. examined CGM data from 117 women, including 89 women with type 1 diabetes, from the first two CGM trials (Murphy et al., Secher et al.), to understand the complex relationships between antenatal glycaemic profiles and large for gestational age infants [21•]. Using a statistical methodology known as functional data analysis, they demonstrated specific maternal glycaemic patterns associated with large for gestational age infants. Lower mean glucose in the first trimester and higher mean glucose in the second and third trimesters were associated with large for gestational age. More specifically, the pattern of glucose associated with large for gestational age infants was lower mid-morning and early evening glucoses in the first trimester, higher early morning and afternoon glucoses in the second trimester and higher evening glucoses in the third trimester. Such detailed information regarding glucose profiles may allow women with diabetes and clinicians to better target insulin delivery with the aim of optimising time in target range and decreasing adverse pregnancy outcomes.

In a different study examining the same 117 women from the same CGM trials (Murphy et al., Secher et al.), Law et al. used CGM data to calculate estimated average glucoses in pregnancy [22•]. Authors noted that estimated average glucoses, as they relate to HbA1c, differ in pregnancy compared to estimates outside of pregnancy; a change in HbA1c in pregnancy represents a smaller change in estimated average glucoses than outside of pregnancy. This study offers patients and clinicians practical guidance in the management of diabetes in pregnancy and recommends aiming for an estimated average glucose of 6.4–6.7 mmol/L in pregnancy.

These studies describe complexities in glycaemic excursions that could not be understood using HbA1c data alone and highlight the potential of CGM to understand how glucose profiles relate to pregnancy outcomes and measures as well as offering clinicians detailed guidance in the care of women with diabetes in pregnancy.

## Insulin Pump Therapy

The physiologic changes throughout pregnancy such as changes in peripheral glucose disposal, insulin pharmacokinetics and decreasing hepatic insulin sensitivity make frequent insulin adjustments necessary [4, 5, 23]. There are also occurrences specific to pregnancy such as antenatal steroid administration, labour and delivery as well as a rapid increase in insulin sensitivity immediately postpartum that require further tailoring of treatment. With these many unique challenges in pregnancy, the use of an insulin pump, also known as continuous subcutaneous insulin infusion, is an attractive option as it facilitates subtle changes in insulin dosing. The insulin pump can be used with capillary glucose monitoring or in conjunction with CGM. When it is used together with CGM, it is referred to as sensor-augmented pump therapy.

Pump therapy outside of pregnancy has been shown to lower HbA1c as well as decrease hypoglycaemia when compared to MDI [24]. However, the recent literature for the use of pump over MDI in pregnancy is inadequate and largely reliant on retrospective observational case series with high risk of bias. The main source of this bias is baseline differences between pump and MDI users; women on the pump tend to be older, have a longer duration of diabetes and are more likely to receive preconception care than women on MDI, making comparisons between the groups challenging [25–27]. The earlier literature reported small randomised studies of varying quality using outdated pumps and MDI from which no conclusions can be drawn.

### Glycaemic Control

Kallas-Koeman et al. performed one of the largest cohort studies including 133 and 218 pregnancies using pump and MDI respectively [25]. They found lower HbA1c levels in insulin pump users than MDI users across all trimesters of pregnancy in both adjusted and unadjusted analyses. Other studies have similarly found lower HbA1c associated with insulin pump use [27–29]. In contrast, a recent Australian study found no significant difference in the HbA1c between women using pumps and MDI [26]. However, women using pumps had a diabetes duration of 20 years compared to 12 years in the MDI group, highlighting the need for larger scale adequately powered randomised controlled trials. A recent systematic review noted there were insufficient data to rule out a difference between the treatment modalities [30].

### Safety of Pumps in Pregnancy

While the benefits remain unclear, most studies suggest that insulin pump use is safe in pregnancy. Pump use does not appear to increase or decrease severe hypoglycaemia episodes, although no studies were specifically powered for this [25–27, 29, 31, 32]. Likewise, for diabetic ketoacidosis, although overall rates are low and even larger sample sizes would be required to detect between group differences [25–27, 31].

### Pregnancy Outcomes

Overall, studies were underpowered to detect difference in most pregnancy outcomes [25–27]. Interestingly, two studies Kallas-Koeman et al. and Neff et al. reported more large for gestational age infants in mothers using insulin pumps (55.0 vs. 39.2% and 36 vs. 20% respectively), with the latter study also reporting more caesarean sections in the pump group (80 vs. 54%) [25, 28]. Another reported more neonatal hypoglycaemia associated with pump use (35 vs. 13%), but again, there were differences in baseline maternal characteristics of women who were and were not offered pumps [32].

### Insulin Pump Use During Labour and Delivery

Tight glycaemic control during labour and delivery is thought to reduce the risk of neonatal hypoglycaemia [33]. Drever et al. examined the safety and efficacy of continuing insulin pump therapy during labour and delivery. In this single-centre retrospective cohort study of 161 pregnancies, they noted that pump therapy appeared safe and was associated with better glycaemic control when compared to women who were switched from their pumps to intravenous insulin infusions [34]. Another cohort study of 65 women found that insulin pump use was safe and performed well (80% of women maintained glucoses within target) [35]. Taken together, these data suggest that the continued use of insulin pump therapy in labour and delivery may be safe and effective in selected patient populations.

## Sensor-Integrated Insulin Delivery

Sensor-integrated insulin delivery is also known as artificial pancreas, (hybrid) closed loop and automated insulin delivery. The three components include a CGM, a single or dual hormonal pump and a computer algorithm to regulate insulin or insulin and glucagon delivery. Sensor-integrated insulin delivery does exactly that; a computer algorithm uses glucose measurements obtained from CGM to determine hormone delivery over time via subcutaneous infusion pump(s). There are many types of sensor-integrated insulin delivery systems. The first commercially available sensor-integrated insulin delivery system, the Medtronic Minimed 670G (Dublin, Ireland), is now available for use in the USA.

A recent meta-analysis of 24 randomised controlled trials (among 585 participants) outside of pregnancy demonstrated that sensor-integrated insulin delivery improved time in range (approximately 70–180 mg/dL) by almost 3 h a day compared to stand-alone pump therapy [36•]. It was also associated with almost 50% less time spent hypoglycaemic (from approximately 5 to 2.5%).

The first trial of sensor-integrated insulin delivery in pregnancy included 16 women with type 1 diabetes [37••]. Women were randomised to a 4-week period of overnight sensor-integrated insulin delivery, compared to sensor-augmented insulin delivery (insulin pump therapy with the use of real-time CGM). Following this, there was a continuation phase of day-and-night sensor-integrated insulin delivery to assess longer term feasibility. Sensor-integrated insulin delivery improved time in target by 15% without increasing hypoglycaemia. Fourteen of the 16 women chose to continue the sensor-integrated insulin delivery system throughout pregnancy. The system continued to perform well as pregnancy progressed, throughout antenatal steroid administration, labour and delivery, hospitalisations and up to 48 h postpartum when it was discontinued.

Farrington et al. highlighted both the advantages and concerns expressed by the women who used the sensor-integrated insulin delivery system in pregnancy [37••, 38•]. While women experienced feelings of improved control, excitement toward the technology and empowerment regarding their diabetes, they also noted concerns about the cumbersome nature of the devices, problems with technical glitches and alarms and concern that with the help of sensor-integrated insulin delivery, they may lose awareness and knowledge regarding diabetes. This study highlights important areas for researchers and clinicians to focus on as the technology becomes more widely available. It also highlights the importance of considering the burdens noted by women using this technology as the technology and evidence advance.

While a commercial hybrid closed loop is available in the USA (the Medtronic Minimed 670G (Dublin, Ireland)), it has challenges in its potential use in pregnancy. It has only been examined in a non-randomised fashion in a non-pregnant population [39]. This study demonstrated a mean time in the target range of 71–180 mg/dL of only 72.2% (SD 8.8); further studies would be needed to see if this closed-loop system can achieve the tight glycaemic targets required to reduce pregnancy complications. Furthermore, glucose targets are pre-set in this commercial device and cannot be lowered by the user or clinician making its use in pregnancy inadequate at present. For now, sensor-integrated insulin delivery has shown promise as a tool in the management of type 1 diabetes in pregnancy. However, this technology must now be studied in larger trials in pregnancy including more diverse populations and settings.

## Conclusions

Emerging technologies in the treatment of type 1 diabetes in pregnancy including CGM, insulin pumps and most recently sensor-integrated insulin delivery show promise in the management of this challenging condition. However, barriers such as cost and the education necessary for each technology must also be considered. The speed of the progress of these technologies offers improvements in accuracy, performance and device burdens associated with their use but also makes it challenging for clinicians to keep up with this ever-changing landscape. An understanding of the current literature is essential, as previously done studies with older devices may not be generalisable to the latest technologies. It also challenges clinicians and women with diabetes to understand and expertly use the various new systems.

Many women with type 1 diabetes put in a tremendous effort in managing their diabetes and may face feelings of concern when their glucose is out of target, pressure to achieve optimal glycaemic control, concern regarding previous pregnancy complications and a desire for a “normal” pregnancy [40, 41]. Despite this, many are unable to achieve guideline-recommended glycaemic targets [6••]. We must find treatments for diabetes that are effective but not all consuming. Technology in the treatment of diabetes may allow us to do so, but there is still much work to be done. It is essential that research continues to keep a fast pace as technology advances and that the perspective of women with type 1 diabetes be taken into consideration as we move forward. The latest evidence suggests that CGM should be offered to all women on intensive insulin therapy. Future research is needed to optimise glucose control before pregnancy and to understand which women are candidates for sensor-integrated insulin delivery.
